# Is DHT Production by 5α-Reductase Friend or Foe in Prostate Cancer?

**DOI:** 10.3389/fonc.2014.00247

**Published:** 2014-09-16

**Authors:** Takeo Kosaka, Akira Miyajima, Mototsugu Oya

**Affiliations:** ^1^Department of Urology, Keio University School of Medicine, Tokyo, Japan

**Keywords:** prostate cancer, androgen receptor, steroidogenesis, 5α-reductase inhibitors, C4-2AT6

## Abstract

The first advance in the history of studies on prostate cancer (PCa) and androgens was the development of treatment with castration and administration of estrogen by Charles B. Huggins, who won the Nobel Prize in Physiology and Medicine. Since then, and for 70 years, androgen deprivation therapy has been the standard therapy for advanced PCa and the center of studies on PCa. However, recent advances have shed light on the relationship between androgens and the development or the progression of PCa. The use of 5AR inhibitors to prevent progression of PCa continues to be widely discussed. Discussion has been fueled by the findings of two large randomized, placebo-controlled trials: the Prostate Cancer Prevention Trial with finasteride and the Reduction by Dutasteride of Prostate Cancer Events trial. Does the development of PCa or progression to castration-resistant PCa depend on dihydrotestosterone (DHT)? Here, we summarize and discuss recent topics of local androgen production of DHT in PCa.

## Introduction

Prostate cancer (PCa) is a malignant tumor that has high morbidity in Europe and the United States, i.e., the first among the male cancers and the second leading cause of death due to cancer in the United States. The morbidity of PCa has also been increasing also in Japan, partly because of the widespread practice of checkup using prostatic-specific antigen (PSA). Thus, huge amounts of research funds are directed to studies given for studies on PCa and this very competitive field has made remarkable advances.

The first progress in the history of studies on PCa and androgens was the development of treatment with castration and administration of estrogen by Charles B. Huggins, who won the Nobel Prize in Physiology and Medicine (1). Since then, and for 70 years, androgen deprivation therapy (ADT) has been the standard therapy for advanced PCa and the center of studies on PCa. However, accumulating evidence has shed light on the relationship between the development and progression of PCa or castration-resistant prostate cancer (CRPC) and androgen–androgen receptor axis (AR axis) (2–7).

On the other hands, the use of 5α-reductase inhibitors (5AR): finasteride or dutasteride among the AR axis targeting drug to prevent development or progression of PCa continues to be widely discussed. Controversies have been fueled by the results of two large randomized, placebo-controlled trials: the Prostate Cancer Prevention Trial (PCPT) with finasteride (8) and the Reduction by Dutasteride of Prostate Cancer Events (REDUCE) trial (9).

Here, we summarize and discuss recent topics of local androgen production and 5α-reductase in PCa.

## AR Axis: Androgen Receptor in Prostate Cancer Tissues

Prostate-specific antigen is a tumor marker commonly used in clinical practice to screen patients with PCa. Consequently, the percentage of men in whom localized PCa has been detected has been increasing; these men are expected to receive complete treatment, including radical prostatectomy and various radiotherapies. However, unfortunately until now, there are many patients with advanced cancer and poorly differentiated carcinoma of high Gleason score and patients who develop a recurrent tumor or metastasis after radical treatment. Most of these patients are treated with ADT (10). However, this therapy has a transient effect, and patients develop hormone-refractory prostate cancer (HRPC), which is resistant to ADT, within several years. AR plays an important role in the advancement of PCa even in patients who undergo castration (11, 12). Since AR is considered to be substantially involved in the pathophysiology of HRPC, this PC is also called CRPC.

The results of studies using cell lines and those on AR expression in patients with PCa showed that AR expression was maintained or enhanced even after ADT in many patients and there is evidence of AR expression in CRPC. Gene expression was analyzed using xenograft models of different PCas and enhanced AR mRNA expression was found to be a common factor of acquired ADT-resistance in many cancer cell strains, showing that cells also respond to a low concentration of androgen (13). On the other hand, reduced AR expression may be controlled epigenetic control by DNA methylation in promotor region may be involved in the mechanism of advanced CRPC (14). It was found that mutations occurred in highly expressed AR and the AR structure was changed downstream of the IL-6 and EGF signaling pathways via STAT3/MAPK-mediated phosphorylation, resulting in AR activation (15–17). It was also found that the expression of co-activators enhancing AR transcriptional activity increased in CRPC, leading to enhanced AR transcriptional activity (7).

## AR Axis: Androgen Production in Prostate Cancer Tissues

Recent progress has revealed intratumoral conversion of adrenal androgens; namely *de novo* steroid synthesis has been proposed as potential causes of PCa progression (18, 19). Results of these studies provide the molecular basis for the inhibition of androgen production and nuclear import of mutated AR in CRPC tissues, leading to actual drug discovery and clinical trials (20–27). The reported high intratumoral testosterone and dihydrotestosterone (DHT) concentrations left in CRPC patients with castrated serum androgen levels have suggested that CRPC maintains a clinically relevant dependence on AR signaling axis. AR activation by androgens converted from adrenal androgens or synthesized intratumorally via the *de novo* route has been proposed as one of the mechanisms of castration resistance (19, 28–31). Some studies using CRPC cancer tissue have investigated intraprostatic testosterone or the active metabolites in quantities, which is thought to be sufficient to stimulate AR-mediated gene expression (32–34). Recent papers have reported that men with a Gleason score of >7 had lower intraprostatic DHT than men with a Gleason score of <6, suggesting that a low-androgen microenvironment predisposes to development or progression for high-grade PCa or CRPC (35–37).

Dihydrotestosterone is the most active androgen, and it was observed that its concentration in PCa tissues did not decrease to the concentration after castration even during ADT and that DHT was produced from adrenal androgen (18, 19). Although 5AR, which is essential for DHT biosynthesis, was identified at the mRNA level in human CRPC metastases (29–31, 38), physiologically relevant 5AR activity in human CRPC has not yet been fully demonstrated.

Recently, authors have just reported a useful experimental model of human CRPC (39–44). We cultured AR positive, PTEN-null, and PSA producing CRPC cell line C4-2 for more than 6 months under androgen ablation media. We were able to establish stable cell line and named it C4-2AT6. These cells seem to harbor aggressive angiogenic properties and elevated phosphorylated Akt expression. These two cell lines may reproduce some part of clinical human CRPC progression and offer an excellent experimental model system with which to investigate complicated biology of CRPC. Using this experimental model, we examined the sequential biosynthesis of DHT from each androgen and were able to find the decreased biosynthesis of DHT in CRPC. To ascertain the 5ARI activity, we co-cultured C4-2 and C4-2AT6 cells with the 13C labeled steroid precursor: 13C-Adione. We examined the sequential biosynthesis of the androgens 13C-T and 13C-DHT, and obtained direct evidence of *de novo* sequential biosynthesis of androgens in both human CRPC cells. CRPC cells were found to express 5AR activity and the activities were thought to be changed under androgen ablation and 5AR activity was not necessarily paralleled by SRD5As expression. To determine whether finasteride and dutasteride have inhibitory effects of the conversion into DHT in CRPC cells, we investigated the concentration of 13C-DHT after treatment with finasteride and dutasteride. LC/MS/MS analysis could not identify 13C-DHT in human CRPC cells. These results indicate that finasteride and dutasteride were able to abrogate the conversion into 13C-DHT in CRPC cells, although finasteride and dutasteride themselves did not have an inhibitory effect on human CRPC (45).

Recently, evidences have shed light on the relationship between AR axis and the PCa development or acquisition of castration resistance (2–5). The use of 5ARIs to prevent progression of PCa is controversial because of the results from recent two large randomized, placebo-controlled PCPT (8) and REDUCE trials (9). The PCPT trial was the first large-scale study to examine the effect of finasteride in relation to PCa development. PCa detected in patients treated with finasteride were of a higher grade than those in patients administered a placebo. High Gleason scores between 7 and 10 were found in 6.4% of the tumors in the finasteride group, compared with only 5.1% of those in the placebo group. The REDUCE trial revealed an overall reduction in the number of PCa patients with a low Gleason score of 5–6 in those receiving dutasteride versus those given a placebo (19.9% compared to 25.1%, respectively). However, during 4-year periods, PCa with high Gleason score of 8–10 were more continual in the dutasteride-treated group than in the placebo group. The FDA analyzed these trials and cited the fact that the obligate increased incidence of tumors with Gleason scores between 8 and 10 by 0.7% with finasteride and by 0.5% with dutasteride. The US Food and Drug Administration’s Oncologic Drugs Advisory Committee voted against recommending 5ARI for the indication to decrease PCa risk in December 2010, because the risk of induction of aggressive PCa outweighed their potential indication for PCa chemoprevention (4). These observations still cannot be fully explained from the view point based on mechanistic analysis. These results suggest that finasteride or dutasteride has little or no effect on more aggressive tumors with high Gleason scores. The decision by FDA not to approve the use of 5ARIs to prevent PCa indicates that further basic and clinical investigations are warranted.

Does the process to CRPC from androgen-dependent PCa depend on DHT produced by 5α-reductase from testosterone? Is it still clinically achievable to treat CRPC using 5ARIs? The efficacies of 5ARIs on metastatic CRPC have not yet been evaluated.

The decreased 5AR activity that we observed in C4-2AT6 cells with the property of human CRPC raised an important critical question: does the death or alive of C4-2AT6 cells depend on DHT? Thus, we examined the effects of DHT on C4-2 and C4-2AT6 cells (40, 43, 45). These human CRPC cells exhibited reduced cell viability when treated with DHT at the dose-dependent manner (45). C4-2 and C4-2AT6 cells exhibit elevated and functional AR expression and produce PSA in response to DHT in a dose-dependent manner; however, C4-2AT6 cells showed significantly lower cell viability. The suppressive effect of DHT on PCa cells is not limited to these *in vitro* results. Some recent clinical studies showed that CRPC could be treated with androgens because of the inhibitory effect of excess androgens (40, 46–49). Accumulating data has represented that AR has a finite ability to bind to T or DHT. However, at higher concentrations, T or DHT has no further additive effect on PCa cell viability when all ARs are bound to T or DHT (40, 46–50). These events are termed as a saturation point. Because of this saturation point, excess DHT may result in the suppression of androgenic-induced proliferation of these cells. We think that CRPC cells have an unknown regulatory system to protect themselves from the excessive androgen with suppressive effects by 5AR activity, although further investigation is needed.

## Conclusion

We reviewed in this article a large number of studies on PCa, which are selected and reviewed from the viewpoint of the authors. For other topics, other valuable articles are available for references.

To resolve many clinical problems and give benefit to the patients, we should actively join basic studies, which lead to multilateral understanding of many valuable basic and clinical studies.

## Conflict of Interest Statement

The authors declare that the research was conducted in the absence of any commercial or financial relationships that could be construed as a potential conflict of interest.
